# Supplementary material for: Research on the coordination mechanism of major industrial project engineering and construction multi-agents based on structural holes theory
Source: PLoS One. 2021 Aug 11;16(8):e0255858. doi: 10.1371/journal.pone.0255858 (PMC8357179; doi:10.1371/journal.pone.0255858)
Supplement: S1 Table — (DOCX) [file pone.0255858.s001.docx]

**S1 Table. List of the edge attribute values between the agents under the EPC model.**

| *i* | *j* | *a_ij_* |
| --- | --- | --- |
| *A* | *B* | 100 |
| *A* | *C* | 100 |
| *A* | *D* | 1000 |
| *A* | *E* | 3000 |
| *A* | *F1* | 500 |
| *A* | *F2* | 400 |
| *A* | *F3* | 300 |
| *A* | *F4* | 250 |
| *A* | *G* | 150 |
| *A* | *H* | 1000 |
| *A* | *I1* | 1000 |
| *A* | *I2* | 100 |
| *A* | *I3* | 300 |
| *A* | *I4* | 100 |
| *A* | *I5* | 200 |
| *A* | *I6* | 800 |
| *A* | *I7* | 500 |
| *A* | *I8* | 2000 |
| *A* | *I9* | 200 |
| *A* | *I10* | 150 |
| *A* | *L1* | 3000 |
| *A* | *L2* | 5000 |
| *A* | *L3* | 1000 |
| *A* | *L4* | 800 |
| *A* | *J1* | 222695 |
| *A* | *J2* | 54138 |
| *A* | *J3* | 87304 |
| *A* | *J4* | 284500 |
| *A* | *J5* | 40878 |
| *A* | *J6* | 39864 |
| *A* | *J7* | 25230 |
| *F1* | *J1* | 100 |
| *F1* | *J2* | 100 |
| *F2* | *J3* | 100 |
| *F2* | *J4* | 100 |
| *F3* | *J5* | 100 |
| *F3* | *J6* | 100 |
| *F4* | *J7* | 100 |
| *A* | *K1* | 800 |
| *A* | *K2* | 1200 |
| *A* | *K3* | 500 |
| *A* | *K4* | 400 |
| *J1* | *K1* | 100 |
| *J2* | *K1* | 100 |
| *J3* | *K2* | 100 |
| *J4* | *K2* | 100 |
| *J5* | *K3* | 100 |
| *J6* | *K4* | 100 |
| *H* | *I1* | 100 |
| *H* | *I2* | 100 |
| *H* | *I3* | 100 |
| *H* | *I4* | 100 |
| *H* | *I5* | 100 |
| *H* | *I6* | 100 |
| *H* | *I7* | 100 |
| *H* | *I8* | 100 |
| *H* | *I9* | 100 |
| *H* | *I10* | 100 |
| *I1* | *L1* | 100 |
| *I2* | *L1* | 100 |
| *I3* | *L1* | 100 |
| *I4* | *L1* | 100 |
| *I5* | *L2* | 100 |
| *I6* | *L2* | 100 |
| *I7* | *L2* | 100 |
| *I8* | *L2* | 100 |
| *I9* | *L3* | 100 |
| *I10* | *L4* | 100 |
| *J1* | *J1K1* | 5000 |
| *J1* | *J1K2* | 800 |
| *J1* | *J1LA1* | 200 |
| *J1* | *J1LA2* | 150 |
| *J1* | *J1LA3* | 140 |
| *J1* | *J1LA4* | 130 |
| *J1* | *J1LA5* | 170 |
| *J1* | *J1LA6* | 1200 |
| *J1* | *J1LA7* | 60 |
| *J1* | *J1LA8* | 800 |
| *J1* | *J1LB1* | 5000 |
| *J1* | *J1LB2* | 400 |
| *J1* | *J1LB3* | 1300 |
| *J1* | *J1LB4* | 1700 |
| *J1* | *J1LB5* | 800 |
| *J1* | *J1LB6* | 780 |
| *J1* | *J1LC1* | 300 |
| *J1* | *J1LC2* | 100 |
| *J1* | *J1LC3* | 100 |
| *J1* | *J1LC4* | 200 |
| *J1* | *J1LC5* | 250 |
| *J1* | *J1LC6* | 140 |
| *J1* | *J1LC7* | 180 |
| *J1* | *J1LC8* | 230 |
| *J1* | *J1LC9* | 610 |
| *J1* | *J1LC10* | 120 |
| *J1* | *J1LC11* | 80 |
| *J1* | *J1LC12* | 90 |
| *J1* | *J1LC13* | 140 |
| *J1* | *J1LD1* | 30 |
| *J1* | *J1LD2* | 20 |
| *J1* | *J1LE1* | 140 |
| *J1* | *J1LF1* | 80 |
| *J1* | *J1LG1* | 800 |
| *J1* | *J1LG2* | 600 |
| *J1* | *J1LG3* | 300 |
| *J1* | *J1LG4* | 200 |
| *J1* | *J1LG5* | 230 |
| *J1* | *J1LH1* | 140 |
| *J1* | *J1LI1* | 200 |
| *J1* | *J1LI2* | 1800 |
| *J1* | *J1LI3* | 500 |
| *J1* | *J1LI4* | 450 |
| *J1* | *J1LI5* | 360 |
| *J1* | *J1LI6* | 230 |
| *J1* | *J1LJ1* | 120 |
| *J1* | *J1LJ2* | 60 |
| *J1* | *J1LJ3* | 45 |
| *J1* | *J1LK1* | 100 |
| *J1* | *J1LL1* | 120 |
| *J1* | *J1LM1* | 300 |
| *J1* | *J1LN1* | 5000 |
| *J1* | *J1LN2* | 2000 |
| *J1* | *J1LN3* | 600 |
| *J1* | *J1LN4* | 370 |
| *J1* | *J1LN5* | 500 |
| *J1* | *J1LN6* | 300 |
| *J1* | *J1LN7* | 200 |
| *J1* | *J1LN8* | 2400 |
| *J1* | *J1LN9* | 430 |
| *J1* | *J1LO1* | 350 |
| *J1* | *J1LO2* | 200 |
| *J1* | *J1LO3* | 200 |
| *J1* | *J1LO4* | 230 |
| *J1* | *J1LP1* | 200 |
| *J1* | *J1LP2* | 260 |
| *J1* | *J1LP3* | 140 |
| *J1* | *J1LQ1* | 80 |
| *J1* | *J1LU1* | 20000 |
| *J1* | *J1LV1* | 130 |
| *J1* | *J1LW1* | 200 |
| *J1* | *J1LX1* | 100 |
| *J1* | *J1LY1* | 500 |
| *J1* | *J1LZ1* | 100 |
| *J1* | *J1L1* | 3500 |
| *J1* | *J1L2* | 80 |
| *J1* | *J1L3* | 136000 |
| *J1* | *J1L4* | 20000 |
| *J1* | *J1L5* | 500 |
| *J1* | *J1L6* | 80 |
| *J1* | *J1L7* | 50 |
| *J2* | *J2K1* | 1200 |
| *J2* | *J2LA1* | 200 |
| *J2* | *J2LA2* | 180 |
| *J2* | *J2LA3* | 320 |
| *J2* | *J2LA4* | 1450 |
| *J2* | *J2LB1* | 800 |
| *J2* | *J2LB2* | 1000 |
| *J2* | *J2LB3* | 780 |
| *J2* | *J2LB4* | 850 |
| *J2* | *J2LB5* | 300 |
| *J2* | *J2LC1* | 900 |
| *J2* | *J2LC2* | 130 |
| *J2* | *J2LC3* | 60 |
| *J2* | *J2LC4* | 80 |
| *J2* | *J2LC5* | 60 |
| *J2* | *J2LD1* | 50 |
| *J2* | *J2LD2* | 20 |
| *J2* | *J2LF1* | 63 |
| *J2* | *J2LG1* | 180 |
| *J2* | *J2LG2* | 120 |
| *J2* | *J2LG3* | 90 |
| *J2* | *J2LG4* | 30 |
| *J2* | *J2LG5* | 70 |
| *J2* | *J2LH1* | 100 |
| *J2* | *J2LI1* | 1010 |
| *J2* | *J2LI2* | 700 |
| *J2* | *J2LI3* | 200 |
| *J2* | *J2LI4* | 200 |
| *J2* | *J2LI5* | 100 |
| *J2* | *J2LI6* | 50 |
| *J2* | *J2LJ1* | 40 |
| *J2* | *J2LJ2* | 10 |
| *J2* | *J2LJ3* | 20 |
| *J2* | *J2LK1* | 80 |
| *J2* | *J2LL1* | 30 |
| *J2* | *J2LM1* | 50 |
| *J2* | *J2LN1* | 1500 |
| *J2* | *J2LN2* | 1300 |
| *J2* | *J2LN3* | 700 |
| *J2* | *J2LN4* | 200 |
| *J2* | *J2LN5* | 100 |
| *J2* | *J2LN6* | 100 |
| *J2* | *J2LN7* | 150 |
| *J2* | *J2LN8* | 1000 |
| *J2* | *J2LN9* | 70 |
| *J2* | *J2LO1* | 150 |
| *J2* | *J2LO2* | 90 |
| *J2* | *J2LO3* | 80 |
| *J2* | *J2LO4* | 100 |
| *J2* | *J2LP1* | 100 |
| *J2* | *J2LP2* | 80 |
| *J2* | *J2LP3* | 85 |
| *J2* | *J2LQ1* | 50 |
| *J2* | *J2LU1* | 1100 |
| *J2* | *J2LV1* | 240 |
| *J2* | *J2LW1* | 40 |
| *J2* | *J2LX1* | 30 |
| *J2* | *J2LZ1* | 40 |
| *J2* | *J2L1* | 1000 |
| *J2* | *J2L2* | 100 |
| *J2* | *J2L3* | 29000 |
| *J2* | *J2L4* | 5000 |
| *J2* | *J2L5* | 130 |
| *J2* | *J2L6* | 50 |
| *J2* | *J2L7* | 30 |
| *J3* | *J3K1* | 2100 |
| *J3* | *J3LA1* | 220 |
| *J3* | *J3LA2* | 800 |
| *J3* | *J3LA3* | 230 |
| *J3* | *J3LA4* | 170 |
| *J3* | *J3LA5* | 210 |
| *J3* | *J3LA6* | 230 |
| *J3* | *J3LA7* | 8200 |
| *J3* | *J3LB1* | 6300 |
| *J3* | *J3LB2* | 680 |
| *J3* | *J3LB3* | 2100 |
| *J3* | *J3LB4* | 1500 |
| *J3* | *J3LB5* | 900 |
| *J3* | *J3LB6* | 1210 |
| *J3* | *J3LB7* | 1500 |
| *J3* | *J3LB8* | 540 |
| *J3* | *J3LC1* | 9000 |
| *J3* | *J3LC2* | 300 |
| *J3* | *J3LC3* | 1000 |
| *J3* | *J3LC4* | 200 |
| *J3* | *J3LC5* | 100 |
| *J3* | *J3LC6* | 80 |
| *J3* | *J3LC7* | 120 |
| *J3* | *J3LC8* | 70 |
| *J3* | *J3LC9* | 200 |
| *J3* | *J3LC10* | 210 |
| *J3* | *J3LC11* | 15 |
| *J3* | *J3LD1* | 10 |
| *J3* | *J3LD2* | 14 |
| *J3* | *J3LE1* | 20 |
| *J3* | *J3LF1* | 30 |
| *J3* | *J3LG1* | 400 |
| *J3* | *J3LG2* | 300 |
| *J3* | *J3LG3* | 80 |
| *J3* | *J3LG4* | 55 |
| *J3* | *J3LG5* | 40 |
| *J3* | *J3LH1* | 110 |
| *J3* | *J3LI1* | 2600 |
| *J3* | *J3LI2* | 2200 |
| *J3* | *J3LI3* | 500 |
| *J3* | *J3LI4* | 400 |
| *J3* | *J3LI5* | 200 |
| *J3* | *J3LI6* | 100 |
| *J3* | *J3LJ1* | 70 |
| *J3* | *J3LJ2* | 30 |
| *J3* | *J3LJ3* | 40 |
| *J3* | *J3LK1* | 50 |
| *J3* | *J3LL1* | 100 |
| *J3* | *J3LM1* | 120 |
| *J3* | *J3LN1* | 3000 |
| *J3* | *J3LN2* | 3200 |
| *J3* | *J3LN3* | 1500 |
| *J3* | *J3LN4* | 1600 |
| *J3* | *J3LN5* | 280 |
| *J3* | *J3LN6* | 300 |
| *J3* | *J3LN7* | 200 |
| *J3* | *J3LN8* | 2400 |
| *J3* | *J3LN9* | 80 |
| *J3* | *J3LO1* | 200 |
| *J3* | *J3LO2* | 150 |
| *J3* | *J3LO3* | 120 |
| *J3* | *J3LO4* | 100 |
| *J3* | *J3LP1* | 220 |
| *J3* | *J3LP2* | 200 |
| *J3* | *J3LP3* | 100 |
| *J3* | *J3LQ1* | 30 |
| *J3* | *J3LU1* | 1800 |
| *J3* | *J3LV1* | 200 |
| *J3* | *J3LW1* | 100 |
| *J3* | *J3LX1* | 90 |
| *J3* | *J3LY1* | 600 |
| *J3* | *J3LZ1* | 100 |
| *J3* | *J3L1* | 1200 |
| *J3* | *J3L2* | 30 |
| *J3* | *J3L3* | 17000 |
| *J3* | *J3L4* | 6500 |
| *J3* | *J3L5* | 220 |
| *J3* | *J3L6* | 100 |
| *J3* | *J3L7* | 30 |
| *J4* | *J4K1* | 3500 |
| *J4* | *J4LA1* | 8000 |
| *J4* | *J4LA2* | 780 |
| *J4* | *J4LA3* | 1500 |
| *J4* | *J4LA4* | 800 |
| *J4* | *J4LA5* | 10000 |
| *J4* | *J4LA6* | 8500 |
| *J4* | *J4LA7* | 500 |
| *J4* | *J4LA8* | 300 |
| *J4* | *J4LB1* | 1000 |
| *J4* | *J4LB2* | 700 |
| *J4* | *J4LB3* | 400 |
| *J4* | *J4LB4* | 3000 |
| *J4* | *J4LB5* | 1000 |
| *J4* | *J4LB6* | 1000 |
| *J4* | *J4LB7* | 600 |
| *J4* | *J4LB8* | 500 |
| *J4* | *J4LB9* | 400 |
| *J4* | *J4LB10* | 650 |
| *J4* | *J4LB11* | 730 |
| *J4* | *J4LB12* | 1500 |
| *J4* | *J4LB13* | 800 |
| *J4* | *J4LB14* | 2500 |
| *J4* | *J4LB15* | 1200 |
| *J4* | *J4LB16* | 1300 |
| *J4* | *J4LC1* | 4500 |
| *J4* | *J4LC2* | 200 |
| *J4* | *J4LC3* | 150 |
| *J4* | *J4LC4* | 200 |
| *J4* | *J4LC5* | 100 |
| *J4* | *J4LC6* | 500 |
| *J4* | *J4LC7* | 600 |
| *J4* | *J4LC8* | 200 |
| *J4* | *J4LC9* | 150 |
| *J4* | *J4LD1* | 25 |
| *J4* | *J4LD2* | 20 |
| *J4* | *J4LE1* | 50 |
| *J4* | *J4LF1* | 100 |
| *J4* | *J4LG1* | 200 |
| *J4* | *J4LG2* | 200 |
| *J4* | *J4LG3* | 80 |
| *J4* | *J4LG4* | 60 |
| *J4* | *J4LG5* | 75 |
| *J4* | *J4LH1* | 100 |
| *J4* | *J4LI1* | 2500 |
| *J4* | *J4LI2* | 3000 |
| *J4* | *J4LI3* | 400 |
| *J4* | *J4LI4* | 450 |
| *J4* | *J4LI5* | 300 |
| *J4* | *J4LI6* | 250 |
| *J4* | *J4LJ1* | 85 |
| *J4* | *J4LJ2* | 60 |
| *J4* | *J4LJ3* | 45 |
| *J4* | *J4LK1* | 100 |
| *J4* | *J4LL1* | 100 |
| *J4* | *J4LM1* | 200 |
| *J4* | *J4LN1* | 5000 |
| *J4* | *J4LN2* | 4500 |
| *J4* | *J4LN3* | 800 |
| *J4* | *J4LN4* | 1000 |
| *J4* | *J4LN5* | 600 |
| *J4* | *J4LN6* | 500 |
| *J4* | *J4LN7* | 800 |
| *J4* | *J4LN8* | 2400 |
| *J4* | *J4LN9* | 200 |
| *J4* | *J4LO1* | 300 |
| *J4* | *J4LO2* | 350 |
| *J4* | *J4LO3* | 200 |
| *J4* | *J4LO4* | 250 |
| *J4* | *J4LP1* | 300 |
| *J4* | *J4LP2* | 250 |
| *J4* | *J4LP3* | 280 |
| *J4* | *J4LQ1* | 230 |
| *J4* | *J4LU1* | 28000 |
| *J4* | *J4LV1* | 300 |
| *J4* | *J4LW1* | 200 |
| *J4* | *J4LX1* | 100 |
| *J4* | *J4LY1* | 800 |
| *J4* | *J4LZ1* | 90 |
| *J4* | *J4L1* | 2000 |
| *J4* | *J4L2* | 150 |
| *J4* | *J4L3* | 8000 |
| *J4* | *J4L4* | 80000 |
| *J4* | *J4L5* | 80000 |
| *J4* | *J4L6* | 500 |
| *J4* | *J4L7* | 180 |
| *J4* | *J4L8* | 60 |
| *J5* | *J5K1* | 1000 |
| *J5* | *J5LA1* | 400 |
| *J5* | *J5LA2* | 500 |
| *J5* | *J5LA3* | 300 |
| *J5* | *J5LA4* | 800 |
| *J5* | *J5LA5* | 1800 |
| *J5* | *J5LA6* | 1600 |
| *J5* | *J5LA7* | 1200 |
| *J5* | *J5LB1* | 600 |
| *J5* | *J5LB2* | 300 |
| *J5* | *J5LB3* | 180 |
| *J5* | *J5LB4* | 250 |
| *J5* | *J5LC1* | 300 |
| *J5* | *J5LC2* | 260 |
| *J5* | *J5LC3* | 100 |
| *J5* | *J5LC4* | 80 |
| *J5* | *J5LC5* | 120 |
| *J5* | *J5LC6* | 30 |
| *J5* | *J5LD1* | 18 |
| *J5* | *J5LD2* | 20 |
| *J5* | *J5LF1* | 30 |
| *J5* | *J5LG1* | 100 |
| *J5* | *J5LG2* | 100 |
| *J5* | *J5LG3* | 80 |
| *J5* | *J5LG4* | 50 |
| *J5* | *J5LG5* | 60 |
| *J5* | *J5LH1* | 50 |
| *J5* | *J5LI1* | 700 |
| *J5* | *J5LI2* | 800 |
| *J5* | *J5LI3* | 200 |
| *J5* | *J5LI4* | 160 |
| *J5* | *J5LI5* | 100 |
| *J5* | *J5LI6* | 100 |
| *J5* | *J5LJ1* | 50 |
| *J5* | *J5LJ2* | 20 |
| *J5* | *J5LJ3* | 30 |
| *J5* | *J5LK1* | 20 |
| *J5* | *J5LL1* | 10 |
| *J5* | *J5LM1* | 40 |
| *J5* | *J5LN1* | 2500 |
| *J5* | *J5LN2* | 2000 |
| *J5* | *J5LN3* | 400 |
| *J5* | *J5LN4* | 600 |
| *J5* | *J5LN5* | 200 |
| *J5* | *J5LN6* | 300 |
| *J5* | *J5LN7* | 400 |
| *J5* | *J5LN8* | 2000 |
| *J5* | *J5LN9* | 100 |
| *J5* | *J5LO1* | 200 |
| *J5* | *J5LO2* | 200 |
| *J5* | *J5LO3* | 150 |
| *J5* | *J5LO4* | 170 |
| *J5* | *J5LP1* | 300 |
| *J5* | *J5LP2* | 200 |
| *J5* | *J5LP3* | 120 |
| *J5* | *J5LQ1* | 80 |
| *J5* | *J5LU1* | 4000 |
| *J5* | *J5LV1* | 200 |
| *J5* | *J5LW1* | 80 |
| *J5* | *J5LX1* | 70 |
| *J5* | *J5LY1* | 50 |
| *J5* | *J5L1* | 500 |
| *J5* | *J5L2* | 50 |
| *J5* | *J5L3* | 13300 |
| *J5* | *J5L5* | 100 |
| *J5* | *J5L6* | 30 |
| *J5* | *J5L7* | 20 |
| *J6* | *J6K1* | 500 |
| *J6* | *J6K2* | 600 |
| *J6* | *J6LA1* | 600 |
| *J6* | *J6LA2* | 400 |
| *J6* | *J6LA3* | 200 |
| *J6* | *J6LA4* | 400 |
| *J6* | *J6LA5* | 700 |
| *J6* | *J6LB1* | 200 |
| *J6* | *J6LB2* | 500 |
| *J6* | *J6LB3* | 400 |
| *J6* | *J6LB4* | 300 |
| *J6* | *J6LC1* | 350 |
| *J6* | *J6LC2* | 400 |
| *J6* | *J6LC3* | 250 |
| *J6* | *J6LC4* | 200 |
| *J6* | *J6LC5* | 200 |
| *J6* | *J6LC6* | 100 |
| *J6* | *J6LC7* | 100 |
| *J6* | *J6LC8* | 70 |
| *J6* | *J6LC9* | 200 |
| *J6* | *J6LC10* | 70 |
| *J6* | *J6LC11* | 50 |
| *J6* | *J6LC12* | 100 |
| *J6* | *J6LC13* | 80 |
| *J6* | *J6LC14* | 100 |
| *J6* | *J6LC15* | 79 |
| *J6* | *J6LC16* | 50 |
| *J6* | *J6LC17* | 100 |
| *J6* | *J6LC18* | 100 |
| *J6* | *J6LC19* | 60 |
| *J6* | *J6LD1* | 20 |
| *J6* | *J6LD2* | 15 |
| *J6* | *J6LG1* | 500 |
| *J6* | *J6LG2* | 500 |
| *J6* | *J6LG3* | 100 |
| *J6* | *J6LG4* | 100 |
| *J6* | *J6LG5* | 50 |
| *J6* | *J6LH1* | 90 |
| *J6* | *J6LI1* | 500 |
| *J6* | *J6LI2* | 500 |
| *J6* | *J6LI3* | 200 |
| *J6* | *J6LI4* | 100 |
| *J6* | *J6LI5* | 100 |
| *J6* | *J6LI6* | 80 |
| *J6* | *J6LJ1* | 80 |
| *J6* | *J6LJ2* | 50 |
| *J6* | *J6LJ3* | 60 |
| *J6* | *J6LK1* | 80 |
| *J6* | *J6LL1* | 60 |
| *J6* | *J6LM1* | 100 |
| *J6* | *J6LN1* | 1500 |
| *J6* | *J6LN2* | 1800 |
| *J6* | *J6LN3* | 300 |
| *J6* | *J6LN4* | 300 |
| *J6* | *J6LN5* | 200 |
| *J6* | *J6LN6* | 150 |
| *J6* | *J6LN7* | 200 |
| *J6* | *J6LN8* | 500 |
| *J6* | *J6LN9* | 600 |
| *J6* | *J6LO1* | 400 |
| *J6* | *J6LO2* | 300 |
| *J6* | *J6LO3* | 100 |
| *J6* | *J6LO4* | 200 |
| *J6* | *J6LP1* | 100 |
| *J6* | *J6LP2* | 100 |
| *J6* | *J6LP3* | 80 |
| *J6* | *J6LQ1* | 60 |
| *J6* | *J6LU1* | 1200 |
| *J6* | *J6LV1* | 100 |
| *J6* | *J6LW1* | 80 |
| *J6* | *J6LX1* | 100 |
| *J6* | *J6LY1* | 80 |
| *J6* | *J6L1* | 400 |
| *J6* | *J6L2* | 40 |
| *J6* | *J6L3* | 8000 |
| *J6* | *J6L4* | 12000 |
| *J6* | *J6L5* | 100 |
| *J6* | *J6L6* | 80 |
| *J6* | *J6L7* | 50 |
| *J7* | *J7K1* | 1000 |
| *J7* | *J7K2* | 1000 |
| *J7* | *J7K3* | 600 |
| *J7* | *J7LD1* | 200 |
| *J7* | *J7LD2* | 100 |
| *J7* | *J7LG1* | 500 |
| *J7* | *J7LG2* | 450 |
| *J7* | *J7LG3* | 350 |
| *J7* | *J7LG4* | 200 |
| *J7* | *J7LG5* | 150 |
| *J7* | *J7LH1* | 100 |
| *J7* | *J7LI1* | 400 |
| *J7* | *J7LI2* | 300 |
| *J7* | *J7LI3* | 100 |
| *J7* | *J7LI4* | 100 |
| *J7* | *J7LI5* | 80 |
| *J7* | *J7LI6* | 60 |
| *J7* | *J7LJ1* | 200 |
| *J7* | *J7LJ2* | 100 |
| *J7* | *J7LJ3* | 60 |
| *J7* | *J7LK1* | 50 |
| *J7* | *J7LL1* | 80 |
| *J7* | *J7LM1* | 100 |
| *J7* | *J7LN1* | 800 |
| *J7* | *J7LN2* | 900 |
| *J7* | *J7LN3* | 200 |
| *J7* | *J7LN4* | 160 |
| *J7* | *J7LN5* | 100 |
| *J7* | *J7LN6* | 120 |
| *J7* | *J7LN7* | 100 |
| *J7* | *J7LN8* | 300 |
| *J7* | *J7LN9* | 100 |
| *J7* | *J7LO1* | 200 |
| *J7* | *J7LO2* | 200 |
| *J7* | *J7LO3* | 150 |
| *J7* | *J7LO4* | 100 |
| *J7* | *J7LP1* | 200 |
| *J7* | *J7LP2* | 200 |
| *J7* | *J7LP3* | 100 |
| *J7* | *J7LQ1* | 700 |
| *J7* | *J7LU1* | 500 |
| *J7* | *J7LV1* | 600 |
| *J7* | *J7LW1* | 500 |
| *J7* | *J7LX1* | 600 |
| *J7* | *J7LY1* | 50 |
| *J7* | *J7L1* | 1000 |
| *J7* | *J7L2* | 100 |
| *J7* | *J7L3* | 5000 |
| *J7* | *J7L4* | 3600 |
| *J7* | *J7L5* | 2000 |
| *J7* | *J7L6* | 300 |
| *J7* | *J7L7* | 20 |
| *J7* | *J7L8* | 50 |
