# Supplementary material for: Research on the coordination mechanism of major industrial project engineering and construction multi-agents based on structural holes theory
Source: PLoS One. 2021 Aug 11;16(8):e0255858. doi: 10.1371/journal.pone.0255858 (PMC8357179; doi:10.1371/journal.pone.0255858)
Supplement: Attachment — Submitted filename: myreview.docx [file pone.0255858.s003.docx]

Dear authors,

Thank you for submitting your paper to the Journal of PLOS ONE. I believe your paper is an informative paper which can be published after a major revision.

**Comments:**

1. It is not clear what the research gap that the paper is addressing. What is the objective of this paper? Please clarify somewhere clearly all your contributions.
2. The literature review is not goal oriented. The process should be as follows:

i) Critical evaluation of the literature; ii) identifying the gap based on this critical evaluation of the literature; iii) proposing your hypothesis to address the identified gap; iv) posing the appropriate and relevant research question based on your proposed hypothesis; and finally explaining your proposed method to answer this research question. Therefore, you will have a systematic way of conducting your research. Right now, the literature review section has no clear objective.

3. An adequate literature review and a clear gap identification have been tried to be conducted. However, authors have ignored some research which has been done in the area. I strongly recommend the authors to provide a more comprehensive literature review in the introduction section. The following papers are recommended:

- Social cohesion, structural holes, and a tale of two measures. Journal of Statistical Physics, 151(3), 745-764.
- Knowledge, networks, and knowledge networks: A review and research agenda. Journal of management, 38(4), 1115-1166.
- Performance evaluation of complex electricity generation systems: A dynamic network-based data envelopment analysis approach. Energy Economics, 104894.
- Complementary or contradictory? The effects of structural holes and status on innovation. Innovation, 20(4), 393-406.

4. A thorough editorial check and English improvement are needed. Please kindly proofread the entire manuscript.
5. The conclusion part is also needed to be revised; which questions are answered, what is the value/originality/contribution of the paper, how the proposed method answers the research questions that previous methods are not able to answer?
6. It feels you need a king of aggregating results somewhere clearer.
7. Please propose and suggest more possible future studies related to the current study.
8. If you can, please make a small comparison between what did you do and what others did before; as a conclusion.
9. The abstract is not deep enough and Is not well prepared. Please try to re-write it better. The problem should be clearly stated and the gap which you are going to address need to be clarified. Simply explain your contributions and key findings.
10. There are some errors in your reference list. Please check and fix the errors.
