# Supplementary material for: Research on the coordination mechanism of major industrial project engineering and construction multi-agents based on structural holes theory
Source: PLoS One. 2021 Aug 11;16(8):e0255858. doi: 10.1371/journal.pone.0255858 (PMC8357179; doi:10.1371/journal.pone.0255858)
Supplement: Attachment — Submitted filename: Response to Reviewers.docx [file pone.0255858.s005.docx]

**Here are the responses to the comments:**

**Reviewer #3:**

1. Research keywords are not carefully selected. It is also necessary to give the reader an adequate explanation after choosing a keyword.

**Reply:** The key words have been revised. The key words are industrial project; major industrial project; multi-agents; coordination mechanism; complex network; structural holes theory. Because the paper is to study the mechanism of the multi-agents relationship of major industrial projects based on the structural holes theory, so the above keywords are selected.

2. What was the lack of available knowledge that led to this research? Research novelty should be clearly stated in the abstract and introduction.

**Reply:** With the development of science and technology, industrial projects tend to be increasingly large-scale and intensive projects. Industrial projects are characterized by a large scale, high investment, complex process system, intensive layout, open-air features, automatic control, high construction technology requirements, numerous design institutes, several suppliers, many construction units, various cooperation units, countless work interface relations, and long construction cycles, and the agent coordination problem of industrial engineering and construction projects is difficult to solve, which requires a new analysis method of the essential characteristics.

The abstract and introduction have been revised, as following:

**Abstract**

Industry is an important pillar of the national economy. Industrial projects are the most complex and difficult projects to control in the construction industry, and major industrial projects are even more complex and difficult to control. Multi-agent coordination is one of the core issues of industrial projects. Based on an analysis of the engineering and construction chains and agent relationships and agent networks of industrial projects, a complex network of the engineering and construction agents of industrial projects is established, and the complex network structural holes theory is applied to study the nonrepeated relationships among agents in industrial projects. Assuming agents are linked through contract relations and the most critical contract index between the agents in the contract amount, through structural hole analysis considering the EPC and PMC model, the aggregate constraint list is obtained, 2D network diagram and 3D network diagram are shown. According to the aggregate constraint value, the EPC contractor with the minimum aggregate constraint value and the project management company with the minimum aggregate constraint value are the critical agent in EPC and PMC model. By analyzing the complex network comprising different models of industrial projects, it is concluded that the characteristics of the agent maintain an advantage in competition, the coordination mechanism of the integration of agent interests, and multi-agent relations are considered to solve the multi-agent coordination problem in major industrial projects.

**1 Introduction**

Major projects include infrastructure or plant projects with a large investment scale, high technical complexity and difficult control process, such as transportation, energy, chemical industry, communications, municipal administration and aerospace projects, which exert an important influence on political, economic, social, science and technology, engineering development, environmental protection, public health, national security and strategic planning aspects.

With the development of science and technology, industrial projects tend to be increasingly large-scale and intensive projects. Industrial projects are characterized by a large scale, high investment, complex process system, intensive layout, open-air features, automatic control, high construction technology requirements, numerous design institutes, several suppliers, many construction units, various cooperation units, countless work interface relations, and long construction cycles, and the agent coordination problem of industrial engineering and construction projects is difficult to solve, which requires a new analysis method of the essential characteristics.

3. It is better to compare the results of this study with other similar studies. Also, the benefits of research are described.

**Reply:** The similar studies have been revised.

**3 Structural holes** [**theory**](javascript:;)

Structural holes are the nonrepeated relationships between two agents. The benefits contributed by the two agents associated with the structural holes to the network are accumulative but not overlapping. There are two indexes to measure structural holes: cohesion and structural allelism. If strong ties occur between two agents, repeated agent relations exist that lack structural holes, and cohesion redundancy follows. A structural hole exists if two of neighbours are not linked to each other. Through these two neighbours, they are connected to different parts of the larger network, and thus have access to different sources of dispersed information. Thus if a firm is to form a new link, closing a structural hole is less valuable than finding a partner to whom none of the current partners is currently connected [19]. The argument is that agents attempt to increase their betweenness centrality. Rodan empirically tested a mediated moderation model that distinguishes between the five different theoretical mechanisms: autonomy, competition, information brokering, opportunity recognition and innovativeness, and the findings suggested that of these five theoretical causal motors, innovativeness plays a key role in linking network structure and network content to performance [20]. Xing et al. analyzed the spreading effect of industrial sectors with complex network model under perspective of econophysics, and the industrial complex network based on input-output tables from WIOD was proposed to be a bridge connecting accurate static quantitative analysis and comparable dynamic one [21]. Latora et al. attempted to reconcile closed and open structures by proposing a new measure, Simmelian brokerage, that captures opportunities of brokerage between otherwise disconnected cohesive groups of contacts, and the implications of our findings for research on social capital and complex networks were discussed [22].

Zhao et al. proposed a novel measure based on Structural Holes and Degree Centrality(SHDC) which combined Structural Hole and Degree Centrality to measure the node influence, and the method used Degree Centrality to make a fast and coarse distinction between the influence of nodes and uses Structure Hole to reflect the impact of topological connections among neighbor nodes, which improved the ability to distinguish the influence of nodes in the low time complexity [23].

The advantages of structural holes are that it emphasizes that structural holes in interpersonal networks can bring advantages in information and other resources to organizations and individuals in that position. If there is no direct connection between the two, and the connection must be formed through the third party, then the third party acting occupies a structural hole in the network of the relationship. Obviously, the structural hole is for the third party.

**References**

1.	Freeman LC. A Set of Measures of Centrality Based on Betweenness. Sociometry. 1977; 40(1):35-41. https://doi.org/10.2307/3033543

2.	Freeman LC. Centrality in social networks : Conceptual clarification. Social Network. 1979; 1(3):215-239. https://doi.org/10.1016/0378-8733(78)90021-7#doilink

3.	Freeman LC, Roeder D, Mulholland RR. Centrality in Social Networks: II. Experimental Results. Social Networks. 1980; 2(2):119-141. https://doi.org/10.1016/0378-8733(79)90002-9

4.	Burt RS. Structural Holes: The Social Structure of Competition. Cambridge MA: Harvard University Press; 1992.

5.	Watts DJ, Strogatz SH. Collective dynamics of 'small-world' networks. Nature. 1998; 393(6684):440-440.

6.	Barabási AL, Albert R.Emergence of scaling in random networks. Science. 1999; 286(5499):509-512.

7.	Rodan S. Structural holes and managerial performance: Identifying the underlying mechanisms. Social Networks. 2010; 32(3):168-179. <https://doi.org/10.1016/j.socnet.2009.11.002>

8.	Latora V, Nicosia V, Panzarasa P. Social Cohesion, Structural Holes, and a Tale of Two Measures. Journal of Statistical Physics. 2012; 151(3):745-764. <https://doi.org/10.1007/s10955-013-0722-z>

9.	Phelps C, Heidl R, Wadhwa A. Knowledge, Networks, and Knowledge Networks: A Review and Research Agenda. Journal of Management. 2012; 38(4):1115-1166. <https://doi.org/10.1177/0149206311432640>

10.	Burt RS. Reinforced structural holes. Social Networks. 2015; 43:149-161. <https://doi.org/10.1016/j.socnet.2015.04.008>

11.	Tohyun K, Kisang P, Eunjung K. Complementary or contradictory? The effects of structural holes and status on innovation. Innovation Management Policy & Practice, 2018; 20(4):393-406. <https://doi.org/10.1080/14479338.2018.1478733>

12.	Deng YJ, Li YQ, Yin RR ,Zhao HY, Liu B. Efficient measurement model for critical nodes based on edge clustering coefficients and edge betweenness. Wireless Networks. 2019; 26(1): 2785-2795. https://doi.org/10.1007/s11276-019-02040-4

13.	Alizadeh R, Beiragh RG, Soltanisehat L, [Soltanzadeh](https://xueshu.baidu.com/s?wd=author%3A%28E%20Soltanzadeh%29%20&tn=SE_baiduxueshu_c1gjeupa&ie=utf-8&sc_f_para=sc_hilight%3Dperson) E， [Lund](https://xueshu.baidu.com/s?wd=author%3A%28PD%20Lund%29%20&tn=SE_baiduxueshu_c1gjeupa&ie=utf-8&sc_f_para=sc_hilight%3Dperson) PD. Performance evaluation of complex electricity generation systems: A dynamic network-based data envelopment analysis approach. Energy Economics. 2020; 104894. <https://doi.org/10.1016/j.eneco.2020.104894>

14.	Han XK, Yan WZ, Lu M. Research on reasoning concerning emergency measures for industrial project scheduling control. Advances in Civil Engineering. 2021; 5595354. https://doi.org/10.1155/2021/5595354

15.	Han XK.WBS-free scheduling method based on database relational model. Int J Syst Assur Eng Manag. 2021; 01106-x. <https://doi.org/10.1007/s13198-021-01106-x>

16.	Han XK, Yan WZ, Lu M. Intelligent Critical Path Computation Algorithm Utilising Ant Colony Optimisation for Complex Project Scheduling. Complexity 2021; 9930113. [https://doi.org/10.1155/2021/9930113](%20https://doi.org/10.1155/2021/9930113)

17.	Nooy WD, Mrvar A, Batagelj V. Exploratory Social Network Analysis with Pajek: Revised and Expanded Edition for Updated Software. Third Edition. New York: Cambridge University Press; 2018.

18.	Mrvar A, Batagelj V. Program for Analysis and Visualization of Large Networks Reference Manual List of commands with short explanation version 5.10. Slovenia: University of Ljubljana; 2020.

19.	Cowan R, Jonard N. Structural holes, innovation and the distribution of ideas. J Econ Interac Coord. 2007; 2: 93–110. <https://doi.org/10.1007/s11403-007-0024-0>

20.	Rodan S. Structural holes and managerial performance: identifying the underlying mechanisms. Social Networks. 2010; 32(3): 168-179. https://doi.org/10.1016/j.socnet.2009.11.002

21.	Xing L, Ye Q, Guan J, Adriana B L. Spreading Effect in Industrial Complex Network Based on Revised Structural Holes Theory. Plos One. 2016, 11(5): e0156270. https://doi.org/10.1371/journal.pone.0156270

22.	Latora V, Nicosia V, Panzarasa P. Social Cohesion, Structural Holes, and a Tale of Two Measures. J Stat Phys. 2013, 151: 745–764. https://doi.org/10.1007/s10955-013-0722-z

23.	Zhao X, Guo S, Wang Y. The Node Influence Analysis in Social Networks Based on Structural Holes and Degree Centrality. 2017 IEEE International Conference on Computational Science and Engineering (CSE) and IEEE International Conference on Embedded and Ubiquitous Computing (EUC). 2017, 708-711. https://doi.org/10.1109/CSE-EUC.2017.137.

4. In conclusion, the direction of research by other researchers in the future is not specified.

**Reply:** Discussion has been added.

This study contributes to the literature by exploratively examining the coordination mechanism of the major industrial project engineering and construction multi-agents. There has been limited research into multi-agents relationship. The influence of organizational characteristics on agent and project performance is a direction of the multi-agents relationship, and how to establish an effective multi-agents social networks is another direction of multi-agents relationship. The prospect of this research is that it can be applied to large, complex and systematic industrial project management, and can be used as a guide for selecting project management model.
